# Rapid LC–MS assay for targeted metabolite quantification by serial injection into isocratic gradients

**DOI:** 10.1007/s00216-022-04384-x

**Published:** 2022-11-28

**Authors:** Ryan A. Groves, Carly C. Y. Chan, Spencer D. Wildman, Daniel B. Gregson, Thomas Rydzak, Ian A. Lewis

**Affiliations:** 1 Department of Biological Sciences, University of Calgary, Calgary, AB T2N 1N4 Canada; 2 Alberta Precision Laboratories, Calgary, AB T2L 2K8 Canada; 3 Department of Pathology and Laboratory Medicine, Cumming School of Medicine, University of Calgary, Calgary, AB T2N 1N4 Canada; 4 Department of Medicine, Cumming School of Medicine, University of Calgary, Calgary, AB T2N 1N4 Canada

**Keywords:** Metabolomics, LC–MS, High-throughput screening, Diagnostics

## Abstract

Liquid chromatography mass spectrometry (LC–MS) has emerged as a mainstream strategy for metabolomics analyses. One advantage of LC–MS is that it can serve both as a biomarker discovery tool and as a platform for clinical diagnostics. Consequently, it offers an exciting opportunity to potentially transition research studies into real-world clinical tools. One important distinction between research versus diagnostics-based applications of LC–MS is throughput. Clinical LC–MS must enable quantitative analyses of target molecules in hundreds or thousands of samples each day. Currently, the throughput of these clinical applications is limited by the chromatographic gradient lengths, which—when analyzing complex metabolomics samples—are difficult to conduct in under ~ 3 min per sample without introducing serious quantitative analysis problems. To address this shortcoming, we developed sequential quantification using isotope dilution (SQUID), an analytical strategy that combines serial sample injections into a continuous isocratic mobile phase to maximize throughput. SQUID uses internal isotope-labelled standards to correct for changes in LC–MS response factors over time. We show that SQUID can detect microbial polyamines in human urine specimens (lower limit of quantification; LLOQ = 106 nM) with less than 0.019 normalized root mean square error. Moreover, we show that samples can be analyzed in as little as 57 s. We propose SQUID as a new, high-throughput LC–MS tool for quantifying small sets of target biomarkers across large cohorts.

**Graphical Abstract:**

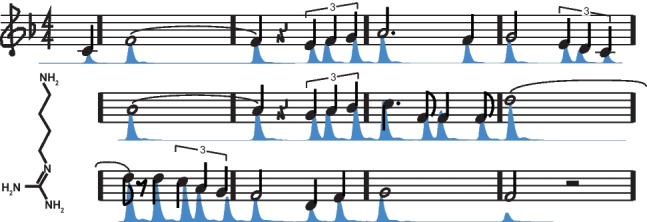

**Supplementary Information:**

The online version contains supplementary material available at 10.1007/s00216-022-04384-x.

## Introduction

Liquid chromatography mass spectrometry (LC–MS) is one of the most common biomarker discovery tools used in metabolomics and has been applied to a broad range of investigations ranging from the mapping of metabolic pathways [1–3], to the tracking of pharmacokinetics in vivo [4], and the discovery of biomarkers that are predictive of disease [5–9]. One of the primary advantages of using LC–MS in these studies is that it provides a direct path between discovery and clinical implementation. Clinical LC–MS is a well-established diagnostic strategy [10, 11] and biomarkers identified in research-oriented studies can be ported into clinical LC–MS diagnostic applications. However, there are significant differences between discovery and clinical applications of LC–MS metabolomics that shape the types of methods that can be employed in clinical settings [12].

Clinical applications of LC–MS are targeted, require absolute quantification, and emphasize sample throughput [10]. These needs are shaped by the regulatory framework that governs in vitro diagnostics and by the need to control costs in clinical reference laboratories [13]. Currently, most clinical applications of LC–MS are completed with triple quadrupole instruments. Throughput on these instruments is typically limited by the number of scans per second or by chromatographic gradient lengths [14, 15]. The high scanning speed of modern instruments [16], in combination with the relatively small number of metabolites that need to be quantified in most clinical assays [13], generally makes chromatography the rate-limiting element in clinical analyses [10].

Chromatography involves balancing trade-offs between metabolite resolution and throughput. Chromatographically resolving metabolites from complex biological samples frequently requires gradients that are 10–20 min long [17]. Although shorter gradients (~ 3 min) [17] and flow injection workflows [18] can be used in some circumstances, both strategies tend to introduce analytical complications that lower the quantitative performance of the LC–MS system [19], largely due to increasing difficulties with ion suppression [20]. Consequently, clinical LC–MS analyses are frequently paired with additional sample handling steps [21–24], such as solid phase extraction, to enable faster LC–MS gradients and reduced matrix effects while concentrating target analytes within samples. Although these supplemental processing steps are effective in maximizing the throughput of the LC–MS system, they add cost and complexity to the overall analytical workflow. Additionally, these processing steps can act as sources of error and as such should be applied judiciously. One well-established approach to correcting for these errors is through isotope dilution—a strategy where a known concentration of an isotope-labelled standard is added to samples and metabolites are then quantified based on the ratio of labelled versus unlabelled metabolites present in extracts [22, 25–27]. In summary, clinical applications of LC–MS that seek to quantify biomarkers from complex biological samples generally need to use long chromatographic gradients or multi-step sample cleanup methods to maximize the throughput of the LC–MS system. Although both strategies are effective [17], a direct LC–MS analysis that enables rapid quantification of target biomarkers without the need for heavy sample processing would be a major benefit to clinical diagnostics. To address this need, we developed the sequential quantification using isotope dilution (SQUID) strategy that combines rapid serial injections to achieve high-throughput and isotope dilution to correct for instrument errors.

SQUID operates under the assumption that target biomarkers in clinical diagnostics generally have a narrow range of chemical properties, and thus can be selectively eluted using carefully calibrated isocratic gradients. In the context of hydrophilic biomarkers, combinations of mobile phases and hydrophilic interaction liquid chromatography (HILIC) stationary phases can be chosen that allow the target metabolites to be eluted while biological salts are retained on the column. This circumstance allows multiple samples to be serially injected into a continuous isocratic solvent flow, wherein the target analytes are eluted in a regular series, but contaminating salts are retained on the column. Since salts are one of the major sources of ion suppression in metabolomics studies [28], this strategy reduces one of the major quantitative problems inherent to flow injection, all while preserving sample throughput [18]. Serial injection strategies have been employed in other studies using different analytical methodologies [29, 30] though their use necessitates additional care with sample normalization. To enable absolute quantification, which is essential for clinical diagnostics, we coupled this serial injection strategy with [U^13^C]-labelled internal standards to enable quantification by isotope dilution, which is an established LC–MS quantitative approach [26]. Herein, we evaluate the efficacy of the SQUID strategy and illustrate its utility in analyzing a cohort of clinical urine specimens for the presence of polyamines, which are an established marker for microbial growth [31].

## Materials and methods

### Chemical reagents and biological samples

Unlabelled agmatine sulfate salt was purchased from Sigma-Aldrich. Due to the absence of a viable commercial vendor, [U-^13^C]agmatine was synthesized on site as previously described in [31]. Briefly, *Escherichia coli* (strain MG1665) was inoculated into M9 minimal media containing 22.2 mM [U-^13^C]glucose and grown overnight at 37 °C (5% CO_2_, 21% O_2_). Overnight saturated cultures were seeded into fresh media and the culture was monitored for glucose levels using a blood glucose monitoring system (Bayer Contour Next) until a level of 5 mM glucose was observed. The culture was then centrifuged for 10 min at 4000 × g and the supernatant was removed, filtered, and adjusted to a pH of 7.0 using a concentrated solution of ammonium bicarbonate. Agmatine from culture was then purified using solid phase extraction. This protocol is described below (scaled to larger column volume proportionally). Resulting eluent was concentrated to 10 × using a vacuum centrifuge at 4 °C and agmatine levels were quantified through a reverse isotope dilution standard curve analyzed by LC–MS using a previously described method [31]. The purity of this solution was assessed to be 98.7% based on ^12^C/^13^C isotope ratio observed across 20 spiked sample injections.

Conversely, [U-^13^C]putrescine was purchased from Cambridge Isotope Laboratories Inc and was reconstituted in 50% methanol for use as an internal standard in bacterial culture experiments.

Patient mid-stream urine samples and clinical isolates of *Escherichia coli* and *Pseudomonas aeruginosa* were acquired from Alberta Precision Laboratories collected under their standardized workflow.

### Isotopic internal standardization of agmatine/putrescine

Both [U-^13^C]agmatine and [U-^13^C]putrescine were used as an internal standard for detecting native agmatine and putrescine in biological samples.

For method validation studies, unlabelled agmatine sulfate salt was used to assess the SQUID method’s performance for agmatine. A standard curve was generated in a 50% methanol/50% urine matrix containing 50 to 50,000 nM ^12^C agmatine and 250 nM [U-^13^C]agmatine (*n* = 4) relative to original urine content. Performance was measured through the lower limit of detection (LLOD) and lower limit of quantification (LLOQ) calculation as well as normalized root mean square error ($NRMSE=\sqrt{\frac{{\Sigma }_{i=1}^{n}({y}_{i}-\widehat{y}{)}^{2}}{n}/\widehat{y}}$). Similarly, [U-^13^C]putrescine was used to assess SQUID’s performance for putrescine.

In order to assess the effectiveness of our isotope normalization strategy, one healthy urine sample was spiked directly with an agmatine standard to a final concentration of 5000 nM ^12^C agmatine and 500 nM [U-^13^C]agmatine.

### Urine sample preparation

Urine samples were prepared as previously described [31]. Briefly, urine samples were fixed 1:1 (v/v) in methanol on site and transferred on dry ice to the Lewis Research Group Laboratory. Samples were stored at − 80 °C until the date of processing and spectral analysis. From these samples, 95 *E. coli*–positive samples and 96 healthy patient samples were identified and selected for further analysis. A volume of 350 µL of each of these samples was combined with 150 µL of an internal standard solution of U^13^C-agmatine constituted in 50% methanol to a final concentration of 250 nM (relative to original urine content) in 96-well plates. These samples were then subjected to solid phase extraction (see below).

### Solid phase extraction

Samples were concentrated using a 96-well Thermo Scientific™ HyperSep™ Silica plate (60108–712, Thermo Fisher Scientific) for solid phase extraction using gravity filtration. This plate was first equilibrated sequentially with water (400 µL) and methanol (400 µL), and then the 500-µL sample solutions were loaded onto the plate. Columns were then washed with methanol (1 mL), water (1 mL), and then methanol containing 0.1% formic acid (250 µL), sequentially. Following this, samples were eluted using 125 µL of water containing 2% formic acid into a 96-well plate. A volume of 25 µL of a concentrated solution of ammonium bicarbonate (pH 8.0) was added to a final concentration of 100 mM in order to partially neutralize the formic acid and raise the solution pH above 3.0, in preparation for LC–MS analysis.

### Microbial culture extract preparation

Microbial sample extracts were cultured and extracted according to a previously described metabolic preference assay [32]. Briefly, three clinical isolates of *E. coli* and *P. aeruginosa* were used to inoculate Mueller–Hinton medium in triplicate which were grown overnight at 37 °C in a humidified incubator (5% CO_2_, 21% O_2_) as seed cultures. Samples were then diluted in fresh medium to 0.5 McFarland and incubated for 4 h under the same atmospheric conditions. Following incubation, samples were centrifuged for 10 min at 4000 × g to pellet bacterial cells and supernatants were removed and fixed at a ratio of 1:1 with methanol. Samples were then diluted tenfold using 50% methanol and spiked with an internal standard of [U-^13^C]putrescine to a final concentration of 250 nM.

### Instrumentation

Chromatographic separation was achieved using a Thermo Scientific™ Vanquish™ UHPLC Integrated biocompatible system (Thermo Fisher Scientific). Heated electrospray ionization was performed using a Thermo Scientific™ Ion Max API Source (Thermo Fisher Scientific). Mass spectral data acquisition was performed on a Thermo Scientific™ Q-ExactiveHF™ mass spectrometer (Thermo Fisher Scientific).

### Chromatographic parameters

All samples were run using a Thermo Scientific™ Syncronis™ ZIC-HILIC column (inner diameter, 2.1 mm; length, 100 mm; particle size, 1.7 µm) with a binary solvent system of 20 mM ammonium formate pH 3.0 in water (solvent A) and 0.1% formic acid in acetonitrile (solvent B). Samples were injected into an isocratic solvent flow of 0.6 mL/min of 86% solvent B at a spacing of 0.95 min (inclusive of sample injection cycle). Sample injection volume used was 2 µL and column compartment temperature was held at 30 °C for all samples. For analysis of positive vs negative urine samples, a more conservative peak spacing of 1.35 min was used to allow for a wider dynamic range of analyte signals while minimizing potential for cross interference between neighbouring peaks. Following all sample batches, column was run at 5% solvent B for 15 min at 0.6 mL/min to elute any accumulated salt before equilibration at 86% solvent B for 5 min.

### Mass spectrometry parameters and data acquisition

All samples were acquired in full scan positive ion mode scanning a mass range of 50–750 m/*z* at 240,000 resolving power. The following source conditions were used: + 3000 V spray voltage, 275 °C capillary temperature, 300 °C vaporizer temperature, 35 arbitrary units (au) sheath gas flow, 15 au auxiliary gas flow, 2 au sweep gas flow. All gas flows were run using nitrogen gas. All spectra were acquired using Thermo Scientific™ Xcalibur™ instrument control software.

### Data analysis

Extracted ion chromatograms for figure production were generated in Xcalibur 4.0.27.19 software using a mass window of (+ / −) 10 ppm. Exact *m*/*z* values used for figure creation and peak integration corresponded to theoretical [M + H]^+^ ion *m*/*z* values and were as follows: ^12^C agmatine, 131.1291 m/*z*; [U-^13^C]agmatine, 136.1459 m/*z*; ^12^C putrescine, 89.1073 m/*z*; [U-^13^C]putrescine, 93.1207 m/*z*. Peak integration was performed using MINT software (publicly accessible at https://github.com/LewisResearchGroup/ms-mint-app/releases/tag/v0.1.7.6) [33]. The MINT software can read LC–MS data output to extract the peak intensities of target metabolites [33]. Data conversion from “.raw” to “.mzXML” files for compatibility with MINT was performed using MSConvert as part of the ProteoWizard software package [34]. Statistical analysis and violin plot creation were performed in R i386 3.5.1 using an in-house developed software package [35]. Data visualization and scientific figure creation were done using Microsoft Excel and Adobe Illustrator.

## Results and discussion

**Fig. 1:**
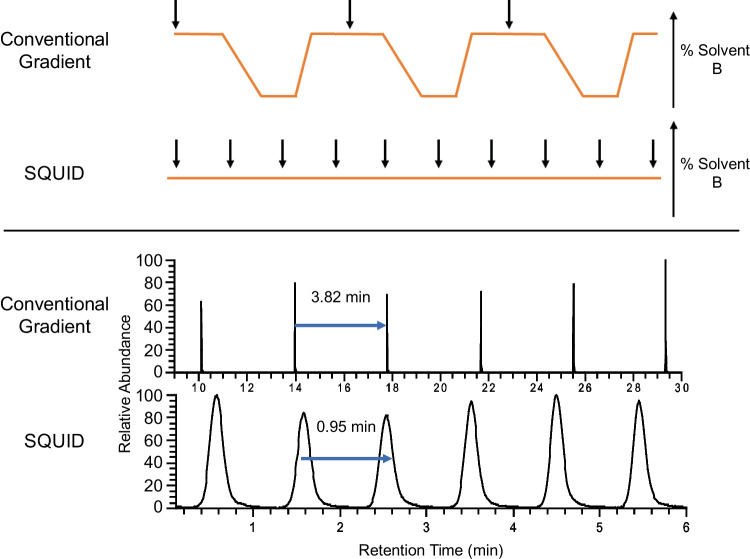
Schematic overview of SQUID. The upper panel demonstrates a conventional injection cycle relative to the SQUID isocratic injection approach. The lower panel demonstrates the resulting chromatographic peak spacing observed when using the conventional versus SQUID approaches. Black arrows indicate the pacing of sample injections under each methodology

SQUID injects samples sequentially into a continuous isocratic flow of solvents (Fig. 1). Target analytes are separated by tuning the mobile phase such that the analytes weakly interact with the column and thereby slowly elute from the column under the isocratic solvent flow. Injection rates are then calibrated to match retention times such that peaks of the target analytes elute at the same cadence as the sample injection rate. We empirically calibrated the mobile phase to optimize the system for analyzing polyamines (putrescine and agmatine) and were able to achieve a 0.95-min spacing between peaks for these compounds (Fig. 1). Given that the autosampler used in this analysis (Thermo Scientific™ Vanquish™ Flex) requires 0.82 min to complete the injection cycle, the SQUID methods presented here are approaching the throughput limits possible on this chromatographic platform.

**Fig. 2:**
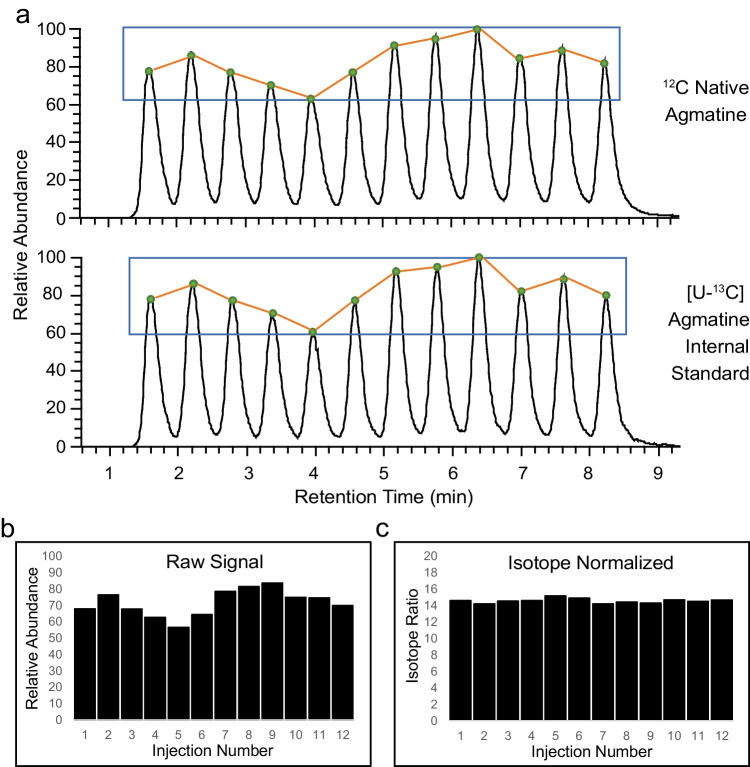
Normalization by isotope ratio resolves differential ion suppression. (**a**) 12 serial sample injections of healthy urine samples spiked with a 5000 nM unlabelled agmatine standard and a 500 nM [U-^13^C]agmatine standard to demonstrate the run-to-run variability in signal intensity across technical replicates. Bar plots show the effect before (**b**) and after (**c**) isotope correction to the internal standard. Isotope ratios were defined as the ^12^C/[U-^13^C]agmatine peak areas observed for each injection

Although the SQUID chromatographic approach captures strong binding molecules (e.g., salts) on the column, metabolites that interact weakly with the stationary phase will elute unpredictably across the serial injection cycle. The co-elution of the target analytes with these other compounds could therefore affect the response factors of the target analytes and thus alter their signal intensities. To address this issue, samples were prepared with a [U-^13^C]-labelled internal standards and native biomarkers were quantified by established isotope dilution methods [10, 36]. As predicted, when one urine sample was injected 12 times consecutively (Fig. 2), the intensity of agmatine was found to be variable (9.35% mean error; normalized root mean square error = 0.108), presumably due to differential co-elution of non-target molecules across the analysis. However, this variability was corrected (1.42% mean error; normalized root mean square error = 0.019) when concentrations of the target analyte were calculated according to the isotope ratio relative to the internal [U-^13^C] standard. In summary, our analyses of technical replicates indicate that SQUID analyses are subject to significant quantitative variability, but this variability can be corrected by computing their concentrations according to isotope dilution methods.

To assess the performance of SQUID as a diagnostic tool, we measured the lower limit of detection (LLOD) and lower limit of quantification (LLOQ) of SQUID when used for the analysis of urinary polyamines. To achieve this, an unlabelled agmatine standard was prepared as a dilution series using a clinical urine specimen containing 250 nM [U-^13^C]agmatine (Supplemental Fig. 1). Using this method, the LLOD of ^12^C agmatine detection was found to be 106 nM and the LLOQ was found to be 353 nM (thresholds defined as 3- and tenfold greater than the noise level, respectively) [37].

**Fig. 3:**
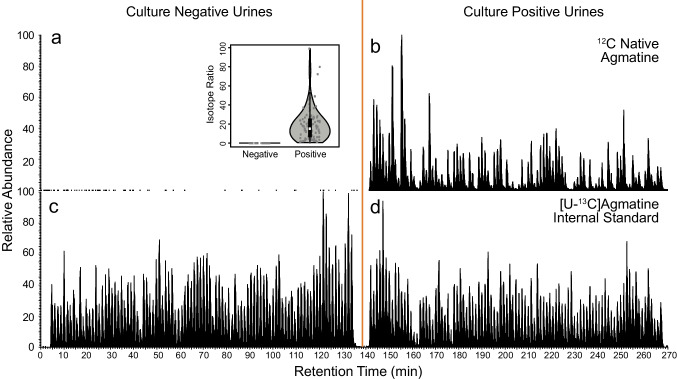
Application of SQUID for detecting polyamines in urine. To illustrate the potential utility of SQUID in high-throughput approaches to detecting microbes, we analyzed 191 human urine specimens provided by Alberta Precision Laboratories by LC–MS. Ninety-five of these samples were identified as culture-positive (≥ 10^7^ CFU/L of *E. coli*) and 96 samples were culture-negative controls (< 10^7^ CFU/mL). Upper quadrants (**a**/**b**) display extracted ion chromatograms for native ^12^C agmatine levels and lower quadrants (**c**/**d**) show the respective internal standard levels

To demonstrate the utility of the SQUID-based approach, a cohort of 191 patient urine samples was analyzed for the presence of agmatine, a microbial polyamine that is produced via the catabolism of arginine [38]. Polyamines have been previously shown to be linked to significant microbial loads in urine [39, 40]. The presence/absence of microbes based on agmatine levels was scored relative to the presence or absence of *E. coli*, as identified by Alberta Precision Laboratories following standard clinical urine culture procedures [41]. As shown in Fig. 3, SQUID could readily distinguish between culture-positive and culture-negative samples. The mean isotope ratio for ^12^C/[U-^13^C]agmatine was 18.7 in positive cultures and 0.005 in negative cultures. Quantitatively, this translates into an average ^12^C agmatine concentration of 2170 nM for culture-positive samples with culture-negative sample levels being below our LLOD (0.581 nM). Moreover, we analyzed the entire cohort of 191 specimens in under 270 min.

**Fig. 4:**
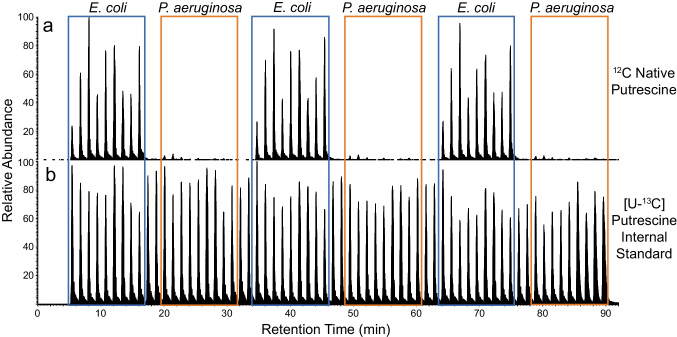
Analysis of in vitro microbial cultures for the presence for putrescine by SQUID. In vitro cultures of two bacterial species, *P. aeruginosa* and *E. coli* (*n* = 9 each), were analyzed by LC–MS across three consecutive technical replicates. SQUID analyses were tuned for the presence of ^12^C (**a**) and [U-^13^C]putrescine (**b**), a microbial polyamine produced by *E. coli* [40]. An internal standard of 500 nM [U-^13^C]putrescine was added to each sample prior to analysis. As expected, putrescine levels could distinguish *P. aeruginosa* from *E. coli* cultures. Blue/orange boxes highlight each technical replicate for the nine biological samples

Clinical diagnostics are only one example of wide range of metabolomics applications that could benefit from the high-throughput, quantitatively robust, targeted analysis made possible via SQUID. Microbial engineering, biofuels research, and antibiotic lead screening are just a few examples of throughput-oriented studies that could benefit from this approach. To illustrate SQUID’s utility for this wider range of potential applications, we analyzed the growth media (Mueller–Hinton) from in vitro cultures of *P. aeruginosa* (*n* = 9) and *E. coli* (*n* = 9) for the presence of putrescine, a microbially produced polyamine that is known to be produced by *E. coli* [40] but not *P. aeruginosa.* As expected, SQUID readily distinguished the *E. coli* from the *P. aeruginosa* cultures based on the presence/absence of putrescine (Fig. 4). This example illustrates that SQUID is applicable to a range of potential biomarkers and sample types and could thus be used in a range of studies where sample throughput is the primary concern.

## Conclusion

Herein, we show that SQUID is a rapid and quantitatively robust LC–MS-based strategy for analyzing small sets of chemically similar catabolites across large cohorts of sample. The methods presented here would allow a single LC–MS platform to analyze over 1000 samples per day and maintain quantitative performance across the cohort. We have illustrated the application of SQUID to two possible metabolomics studies—clinical diagnostics and analyses of in vitro microbial cultures—and have provided a template that will allow SQUID to be adapted to a wide range of throughput-oriented studies.

Although the SQUID methods we present here have obvious benefits regarding instrument efficiency, there are some inherent drawbacks to this strategy that warrant consideration. Firstly, SQUID relies on a precisely calibrated mobile phase to separate target analytes from other compounds and to establish the cadence of injections. Any differences in the chromatographic properties between target analytes will cause the phase of these elution peaks to shift over time and may complicate analyses. Secondly, our analyses illustrate significant sample-to-sample variability in response factors that necessitate the use of isotope-labelled internal standards for each target biomarker. Naturally, this limits the scope of SQUID analyses to metabolites that can either be purchased commercially or can be readily made. In addition, the use of isotope-labelled internal standards limits the dynamic range of metabolites that can be accurately quantified [36]. Thirdly, analyzing SQUID spectra is more challenging than conventional metabolomics since existing software packages are not designed for binning extracted ion chromatograms according to segmented elution windows. In this study, we solved this problem by analyzing spectra directly using our in-house analysis software, Metabolomics Integration Tool (MINT). Large-scale applications of this method would benefit from the use of such a purpose-built software package. In summary, we show that SQUID is a fast and quantitatively robust method for quantifying select target biomarkers that could potentially be applied to a wide range of metabolomics projects.

## Supplementary Information

Below is the link to the electronic supplementary material.

Supplementary file1 (CSV 21 KB)

Supplementary file2 (DOCX 563 KB)
