# Supplementary material for: Rapid LC–MS assay for targeted metabolite quantification by serial injection into isocratic gradients
Source: Anal Bioanal Chem. 2022 Nov 28;415(2):269–76. doi: 10.1007/s00216-022-04384-x (PMC9823034; doi:10.1007/s00216-022-04384-x)
Supplement: Supplementary file 2 — Supplementary file2 (DOCX 563 KB) [file 216_2022_4384_MOESM2_ESM.docx]

**Supplementary Information**

**Rapid LC-MS assay for targeted metabolite quantification by serial injection into isocratic gradients**

Authors: Ryan A Groves^1^, Carly CY Chan^1^, Spencer D Wildman^1^, Daniel B Gregson^2,3,4^, Thomas Rydzak^1^, Ian A Lewis^1^

^1^Department of Biological Science, University of Calgary, Calgary, AB T2N 1N4, Canada

^2^Alberta Precision Laboratories, Calgary, AB, T2L 2K8, Canada

^3^University of Calgary, Cumming School of Medicine, Department of Pathology and Laboratory Medicine, Calgary, T2N 1N4, Canada

^4^University of Calgary, Cumming School of Medicine, Department of Medicine, Calgary, T2N 1N4, Canada

**
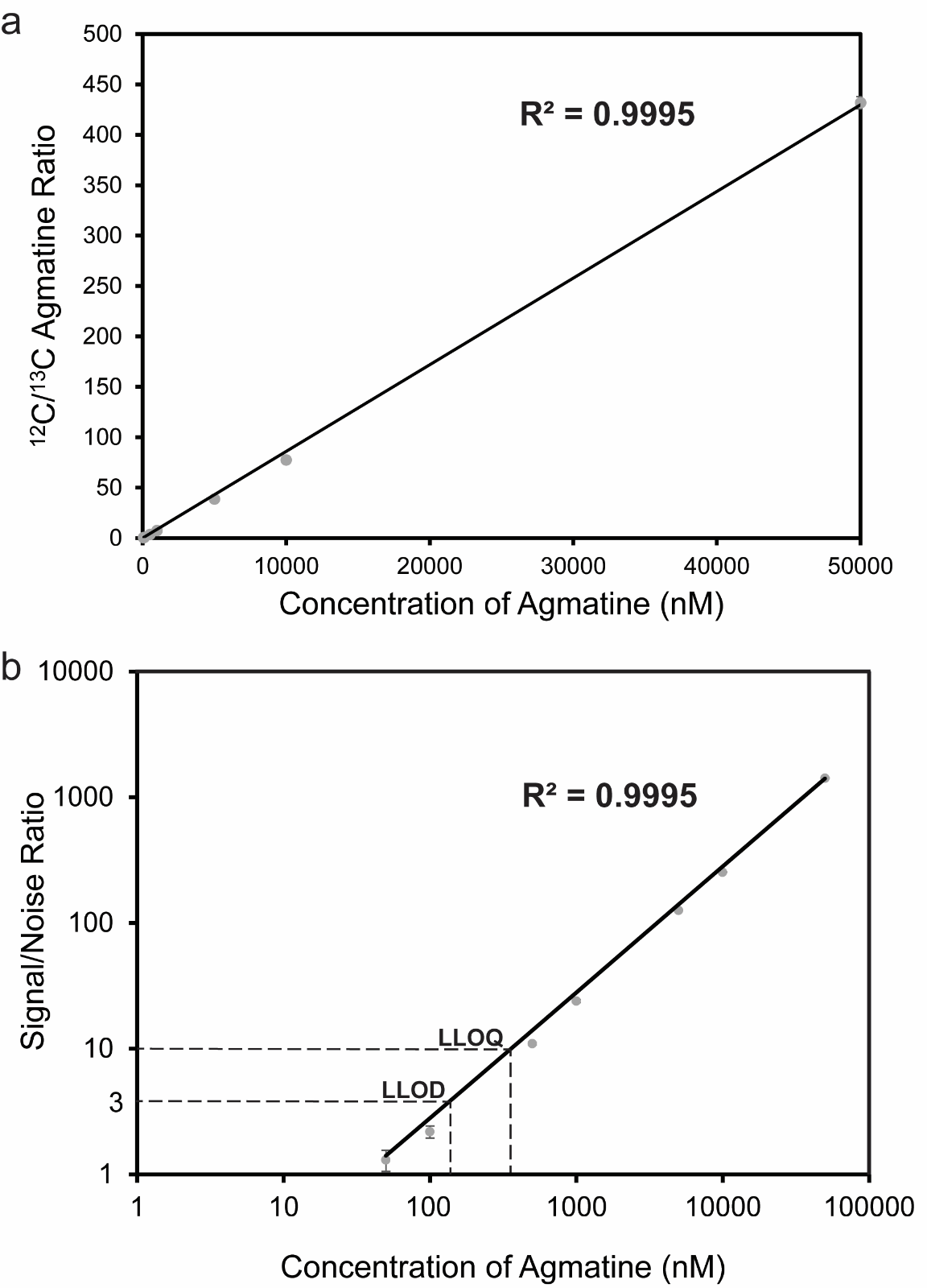
**

**Supplemental Fig. 1 Calibration reference samples prepared by adding agmatine standards to urine. (a)** ^12^C agmatine standards with concentrations ranging from 50 to 50,000 nM (n = 4) were added to 50% urine samples containing 250 nM of [U-^13^C] agmatine. Samples were analysed by SQUID and ^12^C/[U-^13^C]agmatine signal intensity ratios were plotted versus concentration. **(b)** Signal/noise ratio was plotted for each standard curve point for the purpose of calculating LLOD and LLOQ values. The lower limit of detection (LLOD = 106 nM) and lower limit of quantification (LLOQ = 353 nM) were defined as the sample points that were three and 10 times greater than the noise threshold. Error bars represent standard error.


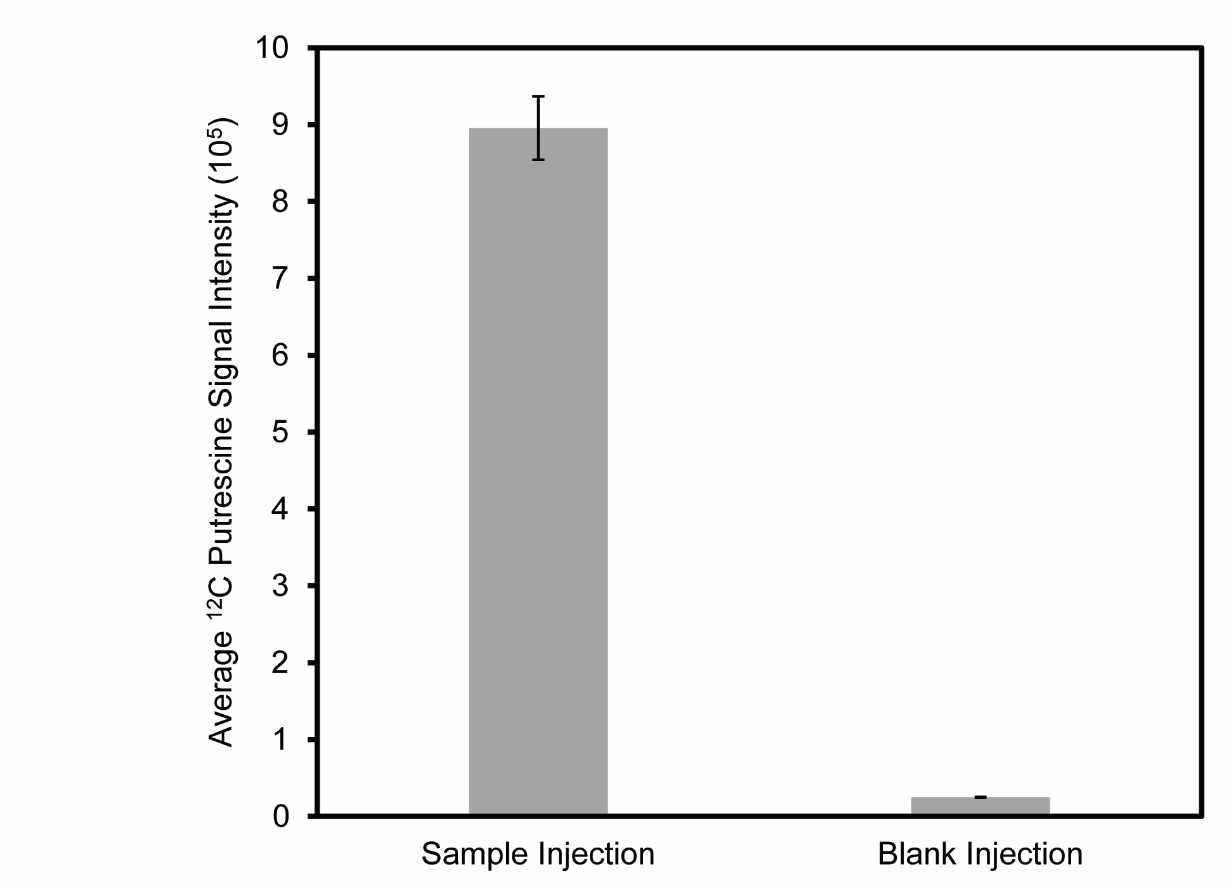


**Supplemental Fig. 2. Carry-over performance analysis of SQUID.** *E. coli* cultures (n = 9) were sequentially injected followed by a blank sample to assess carry over. The entire sequence was repeated three times to assess average carry-over between high-abundance samples and follow-up samples. The blank signals (which included noise) were 2.8% of the intensity of the agmatine signals.


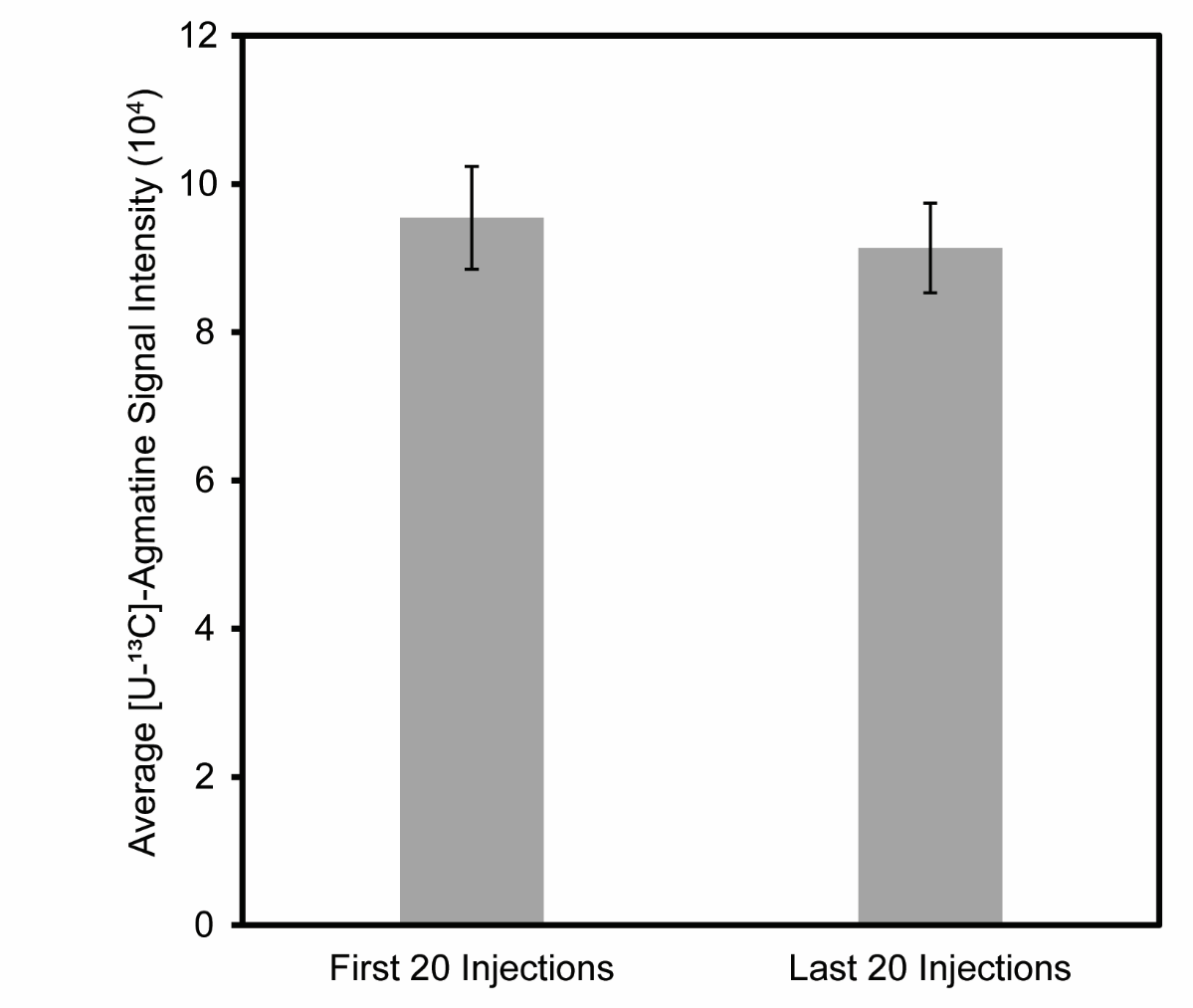


**Supplemental Fig. 3. Signal stability of [U-^13^C]agmatine when analyzed by SQUID.** A total of 191 urine samples spiked with 250 nm [U-^13^C]agmatine were consecutively analyzed with SQUID. The signal intensity of [U-^13^C]agmatine in first 20 sample injections were compared to the last 20 sample injections in the LC-MS run in order to assess signal intensity drift.
